# Delir im psychiatrischen Konsildienst

**DOI:** 10.1007/s00115-025-01836-4

**Published:** 2025-05-30

**Authors:** Eike Ahlers

**Affiliations:** 1 Johanniter-Krankenhaus Treuenbrietzen, Johanniter-Straße 1, 14929 Treuenbrietzen, Deutschland; 2 Klinik für Psychiatrie und Psychotherapie, Charité Universitätsmedizin Berlin, Berlin, Deutschland

**Keywords:** Delirmanagement, Psychiatrischer Konsil- und Liaisondienst, Kognitive Störungen, Demenz, Nichtpharmakologische Maßnahmen, Delirium management, Psychiatric consultation and liaison service, Cognitive disorders, Dementia, Nonpharmacological techniques

## Abstract

**Hintergrund:**

Anfragen zu Delirien stellen eine sehr häufige Fragestellung im psychiatrischen Konsil- und Liaisondienst (CL) dar.

**Fragestellung:**

Welche praktischen Vorgehensweisen lassen sich entsprechend aktuellen Empfehlungen ableiten?

**Material und Methode:**

Auswertung aktueller Leitlinien und Literaturempfehlungen sowie Erfahrungen der interdisziplinären Planung von Delirmanagement an verschiedenen Krankenhäusern und Anmerkungen der Teilnehmenden in Workshops des Autors.

**Ergebnisse:**

Delirien werden als große Belastung bei der Behandlung schwer Kranker über alle Disziplinen angesehen. Es existieren medizinische nationale und internationale Leitlinienempfehlung, welche übereinstimmend ein institutionelles Delirmanagement empfehlen, welches für die jeweilige Abteilung ein standardisiertes Vorgehen zur Risikominimierung und Prävention von Delirien verfolgt. Gerade bei hohem Alter kann kognitiven Defiziten Vorschub geleistet werden. Pathophysiologische Modellvorstellungen können die Behandlungsindikation unterstützen. In der Behandlung zeigen sich vor allem nichtpharmakologische Maßnahmen effektiv, pharmakologische Schritte sollen gut abgewogen und differenziert erfolgen. Die Kommunikation mit Betroffenen und Angehörigen und die Überleitung bei Entlassung oder Verlegung sollte explizit mit einbezogen werden.

**Schlussfolgerungen:**

Im CL können Schritte der Diagnostik und Behandlung und des abteilungsspezifischen, multiprofessionellen Delirmanagements unterstützt werden, vor allem die der differenzierten Psychopharmakotherapie. Weitere Anstrengung bei der Implementierung eines wirksamen Delirmanagements sind auch institutionell sinnvoll möglich, um Prognose und Lebensqualität der Betroffenen zu verbessern.

## Abstract (en)

**Background:**

Inquiries about delirium are a very common request in psychiatric consultation and liaison services (CL).

**Objectives:**

What practical procedures can be derived from current recommendations?

**Methods:**

Evaluation of current guidelines and literature recommendations, as well as of the experiences in interdisciplinary planning of delirium management at various hospitals and of comments of participants in the author’s workshops.

**Results:**

Delirium is seen as a major burden in the treatment of seriously ill patients across all medical disciplines. There are national and international medical guideline recommendations that unanimously recommend institutional delirium management, which follows a standardized approach for the respective department to minimize risk and prevent delirium. Cognitive deficits can follow delirium, especially in old age. Pathophysiological models can support the indication for treatment. Nonpharmacological techniques are effective; pharmacological steps should be carefully considered. Communication with those affected and their relatives and the transition upon discharge or transfer should be explicitly included.

**Conclusions:**

The CL can support steps and department-specific optimization of multiprofessional and delirium management, especially differential psychopharmacotherapy. Further efforts in the implementation of effective delirium management are also possible institutionally in order to improve the prognosis and quality of life of those affected.

Anfragen zu Delirien sind im psychiatrischen Konsildienst sehr häufig. Hier soll auf die multiprofessionelle Zusammenarbeit in der Behandlung und auch auf die Verzahnung mit gerontopsychiatrischen Fragestellungen nach aktuellem Kenntnisstand eingegangen werden. Maßgeblich ist, die Prognose und Lebensqualität von Betroffenen positiv zu beeinflussen. Im interdisziplinären Delirmanagement sollte entlang eines Verständnisses von Delirien als Störungsbilder und in mehreren ineinandergreifenden Phasen (Prävention, Diagnostik, Behandlung und Nachsorge) vorgegangen werden.

## Delir im Krankenhaus

Ein Delir oder Delirium ist eines der häufigsten ZNS(Zentralnervensystem)-Syndrome in Krankenhäusern [1]. Es ist genauso ein klassisches, sehr gut beschriebenes psychiatrisches Syndrom. Es ist phänomenologisch gesehen komplex und mitunter nicht leicht zu erkennen oder differenzialdiagnostisch abzugrenzen. Gerade Verhaltensstörungen mit Agitiertheit, kognitiven Defiziten und psychotischen Symptomen können auch bei demenziellen Syndromen auftreten (vgl. [2]). Es gilt als Zeichen schwerer Erkrankung und Konsequenzen sind u. U. eine deutliche Verschlechterung der Prognose. Kognitiven Defiziten kann dabei Vorschub geleistet werden. Über die medizinischen Auswirkungen hinaus wird eine deutliche Erhöhung der Behandlungskosten angenommen (39 % bei ITS[Intensivstation]-Behandlung [3]).

Es existieren zunehmend mehr spezifische Fachliteratur und darüber hinaus konkrete Leitlinienempfehlungen (s. Infobox 1). Außerdem entstehen immer mehr Initiativen und Projekte zum Delirmanagement im Krankenhaus (u. a. Gemeinsamer Bundesausschuss [G-BA]/Institut für Qualitätssicherung und Transparenz im Gesundheitswesen [IQTIQ] zur Prävention postoperativer Delirien [4–7]).

### Delir im psychiatrischen Konsildienst

Konsilanfragen zu Delirien sind sehr häufig und kommen aus fast allen medizinischen Disziplinen [8], insbesondere aus Rettungsstellen und Intensivstationen. Nach Verlegung auf Normalstationen im Tagdienst kommt es aber auch nicht selten zu abendlichen Exazerbationen mit teils erheblichen auch gefährdenden Fehlhandlungen. Unkenntnis über die Symptome und deren Behandlung verzögert das Erkennen oder erschwert die Prävention. Dementsprechend oft sind hyperaktiv delirante Syndrome mit Fragen nach Akutbehandlung und Übernahme führend. Der Natur des Störungsbildes entspräche aber eher ein jeweils abteilungsspezifischer Umgang mit deliranten Symptomen zur Prävention schwerer Exazerbation. Die Erfahrung im psychiatrischen Konsil- und Liaisondienst (CL) zeigt auch, dass eine Unterstützung des Delirmanagements sinnvoll ist, um die abteilungsspezifischen Abläufe zu verbessern [9, 10].

Der Beitrag im CL kann unterschiedliche Schwerpunkte haben (Tab. 1). Meist ist es primär wichtig ein Delir zu diagnostizieren und auf Ursachensuche und Behandlung zu fokussieren. Oft ist sinnvoll, das Behandlungsteam zu bestärken, die nichtpharmakologischen, pflegerischen Maßnahmen, trotz der Verhaltensstörungen der betroffenen Patienten, anzuwenden und diese ärztlich zu koordinieren und anzuordnen. Dies sollte auch dokumentiert und adäquat kommuniziert werden. Gleichzeitig ist speziell die psychopharmakologische Expertise gefragt, um für den Einzelfall eine differenzierte Pharmakotherapie mit Angabe von Dosierungen vorzuschlagen. Im Verlauf ist für Folgen und Anforderungen über die Entlassung hinaus zu sensibilisieren. Außerdem können in den Abteilungen und Teams Verfahren von der Risikoerfassung bis zur Behandlung und Überleitung unterstützt werden.

**Tab. 1:** Praxisbezogene Darstellung der Aufgaben des psychiatrischen Konsil- und Liaisondienstes bei Delirien

Checkliste für die Praxis: Aufgaben im psychiatrischen Konsil- und Liaisondienst bei Delir
**1. Detektion/Diagnostik**
*Untersuchung, Anamnese, validierte Tests, modifizierbare Risikofaktoren einbeziehen*
Einzelne Schritte: psychopathologische und fokussierte medizinische Untersuchung, Anamnese, settingspezifische (altersmedizinisch 1 Tag nach Aufnahme und im Verlauf, ITS möglichst pro Schicht) strukturierte Anwendung diagnostischer Skalen (4AT, Nu-DESC, CAM-ICU, DOS u. v. m.)
Exploration, Fadenprobe, körperliche Untersuchung; Ursache, Risikofaktoren, Differenzialdiagnosen; Hinweise auf Delirursachen; längsschnittliche Anzeichen für Risikofaktoren (modifizierbar?) und für Demenz
**2. Behandlung**
*Nichtpharmakologische Maßnahmen*
Flüssigkeitsmanagement, Ernährung, Mobilisierung, Ausgleich sensorischer Defizite, Schlafhygiene, Einbezug Angehöriger u. v. m.
Vor allem Reorientierung tagsüber und Schlafförderung nachts, einfache Teammaßnahmen propagieren
*Pharmakotherapie*
Einzelne Schritte: differenziert empfehlen, antidelirant symptombezogen, schlafunterstützend (Rhythmisierung fördern), Sedierung (wo klinisch möglich tiefe Sedierung vermeiden, insbesondere unter Einsatz von Benzodiazepinen und auch α2-Agonisten) und Analgesie optimieren, Polypharmazie reduzieren
Anmerkungen: möglicherweise Prävention erwägen; Antipsychotika niedrig dosiert oder auf ITS α2-Agonisten; andere Pharmaka erwägen, Bedarfsmedikation und Alternativen konkret benennen, Medikationsliste checken (Sedativa, Anticholinergika ggf. absetzen)
**3. Überleitung**
*Entlassbefund, -dokumentation; weitere Empfehlungen*
Einzelne Schritte: Pharmakotherapie, anhaltende kognitive Beeinträchtigungen oder Verdacht auf POCD, PTSD-assoziierte Symptome
Anmerkungen: Medikamente absetzen nicht vergessen oder Indikation und Verlaufsbeobachtung empfehlen, Versorgungsbedarf identifizieren, Risiko für erneute Delirien markieren
**4. Delirmanagement**
*Arbeit der Abteilung unterstützen*
Einzelne Schritte: abteilungsspezifische, häufige Konsilfragestellungen identifizieren; SOPs ermutigen, multiprofessionell beraten
Anmerkungen: Konsilempfehlungen immer den Teams (Pflege, Ärzte, Therapeuten) rückmelden, gegenfragen, systematische Aufnahme Screening und Beobachtung über das Schichtsystem ermutigen, Angehörige mit einbeziehen
**5. Kommunikation**
*Kommunikation mit Betroffenen und Angehörigen unterstützen*
Einzelne Schritte: wenn gewünscht Patienten und Angehörige entlasten, beraten, Teamkommunikation fördern
Anmerkungen: Teilnahme an Visiten oder Angehörigengesprächen, Kommunikationsstil der Teams erfragen und Empfehlungen (wertfrei, zugewandt-stressreduzierend) rückmelden bei herausfordernder Psychopathologie des Delirs und Stigmatisierung; pathophysiologische Zusammenhänge mit deliranter Wesensveränderung besprechen

*CAM-ICU* Confusion Assessment Method, *DOS* Delirium Observation Screening Scale, *ITS* Intensivstation, *Nu-DESC* Nursing Delirium Screening Scale, *POCD* postoperatives kognitives Defizit, *PTSD* posttraumatische Belastungsstörung, *4AT* 4-Assessment Test for Delirium, *SOPs* Standard Operating Procedures

### Herausforderung durch heterogene Klinik

Ein Delir tritt subakut oder akut auf und fluktuiert meist zirkadian. Die Dauer kann stark variieren. Es bestehen unterschiedliche medizinische oder organische Ursachen, welche zu Symptomen wie Bewusstseinsstörungen, allgemeinen kognitiven Beeinträchtigungen, Desorientierung, Wahrnehmungsstörungen, Aufmerksamkeitsdefiziten, Veränderungen der Psychomotorik und gestörtem Schlaf-Wach-Rhythmus führen und in der Schwere der Symptome sehr unterschiedlich sein können [1]. Weitere psychopathologische Beeinträchtigungen können aber auch im Vordergrund in einer querschnittlichen Untersuchung erscheinen, z. B. sehr unterschiedliche Affektstörungen und -äußerungen (Ratlosigkeit bis zu Erregung, negativistischen und Suizidäußerungen). Diese sind in medizinischen Disziplinen außerhalb der Psychiatrie mitunter schwierig zuzuordnen. Sehr unterschiedliche Anfragen an psychiatrische Konsildienste (von Fragen nach Behandlung von „Verwirrung“, über Fragen nach Suizidalität und Depressivität bis hin zu Fragen nach Übernahme bei hypermotorischen und gefährdenden Fehlhandlungen) kennzeichnen so den Klinikalltag.

### Prognoseverschlechterungen begründet Fokus auf Prävention und Behandlung

Delirante Syndrome, aber auch subsyndromale [11] Zustände gehen jeweils mit einer Prognoseverschlechterung über alle Settings im Krankenhaus einher [12]. Gerade die Zunahme der Mortalität nach Delirien gilt für ältere Patienten, für Patienten auf ITS und beatmete Patienten. Komplikationen sind v. a. akute Atemnotsyndrome, nosokomiale Pneumonie, kardiopulmonales Ödem, Reintubation, Selbstextubation, Katheterentfernung, Herzrhythmusstörungen und längere Beatmungszeiten [13].

### Akzeleration demenzieller Entwicklung bei Delirien und reziproke Effekte

Eine an vielen Stellen beschriebene und bekannte klinische Erfahrung ist, dass bestehende kognitive Defizite, einschließlich Demenz, die Schwelle zur Entwicklung eines Deliriums senken. Daten deuten bei älteren Patienten auf eine signifikante Beschleunigung kognitiven Abbaus bei Alzheimer-Krankheit nach deliranten Symptomen hin [14]. Delirprävention wird dementsprechend in der Versorgung Älterer und auch in der Longevity-Forschung propagiert [15].

Ein erheblicher Anteil der Patienten, die ein Delir überleben, leidet anschließend unter langfristigen kognitiven Beeinträchtigungen [16, 17]. Eine mögliche Erklärung für die Beziehung zwischen Delirium und kognitiver Leistungsfähigkeit könnte in einem Zusammenhang zwischen längerer Delirdauer und stärkerer Hirnatrophie liegen [18].

### PTBS-assoziierte Symptome als Folge von Delirien

Ein weiterer oft wenig und vor allem wenig systematisch erfasster Folgeeffekt deliranter Zustände können PTBS(posttraumatische Belastungsstörung)-assoziierte Beschwerden sein. Im Konsildienst zeigt die Erfahrung, dass Betroffene und Angehörige stressende Erfahrungen durchaus in der Behandlung ansprechen. Die Pflege erfährt von Ängsten und Problemen der Patienten, z. B. stattgehabte Halluzinationen zu verstehen und einzuordnen [19, 20]. Auch hier kann eine Aufklärung und Stärkung delirgerechter Kommunikationsstile (unvoreingenommen und emotional zugewandt [21]) als Aufgabe des CL erwogen werden.

### Befund bei Entlassung und Überleitung

Eine vollständige Genesung ist zum Zeitpunkt der Entlassung oder Verlegung bei komplexen Fällen eher selten. Eine Behandlung sollte deshalb effektiv übergeleitet werden. Auf die Überleitung des Befundes und der Empfehlungen (Verlaufsmonitoring, Absetzen von Antipsychotika, Unterstützung im Alltag u. a.) sollte explizit hingewiesen werden [22].

## Pathophysiologie von Delirien

**Abb. 1:**
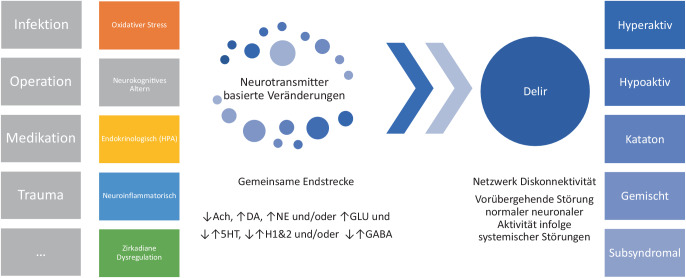
Neuropathogenese als systemisches Krankheitsgeschehen und Ursache heterogener Klinik bei Delirien. *Ach* Acetylcholin, *DA* Dopamin, *NE* Noradrenalin, *GLU* Glutamat, *5HT* Serotonin, *H1&2* Histaminrezeptoren, *GABA *γ‑Aminobuttersäure, *HPA* Hypothalamus-Hypophyse-Nebennierenrinden

Pathophysiologisch gesehen ist ein Delir eine neurologische Funktionsstörung und ein Verhaltenssyndrom, das durch eine vorübergehende Störung der normalen neuronalen Aktivität infolge von Störungen der systemischen Physiologie verursacht wird (Abb. 1).

Auch wenn noch keine lückenlos nachgewiesene pathophysiologische Vorstellung präsentiert ist, konfluieren die unterschiedlichen Ideen zu einer gemeinsamen Endstrecke aus Neurotransmitterveränderungen und Netzwerkausfällen, was gut zu den unterschiedlichen und wechselhaften (psychomotorischen sowie zirkadianen) klinischen Bildern passen könnte. Gerade im CL, wenn man effizient mit verschiedenen medizinischen Teams die Behandlung unterstützen möchte, sind pathophysiologische Modellvorstellungen eine Chance, zu motivieren, die Ursache zu suchen und spezifische Behandlung einzuleiten. Besonders wertvoll erscheint die Kenntnis auch ob der Möglichkeit symptomorientiert pharmakologisch zu behandeln, was in der Regel die spezifische Anfrage im Delirmanagement an das psychiatrische Konsil ist.

Unterschiedliche Ursachen bei schwerer Erkrankung führen zu verschiedenen systemischen Veränderungen (z. B. aufgrund von Veränderungen des oxidativen Stoffwechsels und Hypoxie [z. B. intraoperativ], Alterung [insbesondere „Mechanismen neuronalen Alterns“, Beeinträchtigung der cholinergen Neurotransmission], endokrinen Störungen [Hypothalamus-Hypophyse-Nebennierenrinden-Achsen-Aktivität und bei Veränderungen im Glukokortikoidstoffwechsel], Neuroinflammation [23], Veränderungen des Schlaf-Wach-Musters und im melatonergen System), welche in der Endstrecke zu veränderter Neurotransmission (v. a. Exzesse aktivierender Neurotransmitter und Beeinträchtigungen der cholinergen Neurotransmission) und damit zu unterschiedlicher und fluktuierender Netzwerkkonnektivität führen. Es resultiert ein gerade kognitiv und psychomotorisch heterogenes klinisches Bild [24].

## Prävention, Behandlung und Delirmanagement

Im Management von Delirien ist eine Zuweisung spezifischer Aufgaben im multiprofessionellen Team (mit Ärzten, der Pflege und den Spezialtherapeuten wie Physio‑/Ergotherapie, Atemtherapie u. v. m.) und Zusammenarbeit des primären Behandlungsteams und ggf. seiner Berater (einschließlich des CL) entscheidend. Mehrere, teils überlappende, Schritte sollten in allen medizinischen Abteilungen berücksichtigt werden:

1. Erkennen von Risikopatienten,
2. Implementierung präventiver Maßnahmen (pharmakologische und v. a. nichtpharmakologische),
3. verbesserte regelmäßige Überwachung und strukturiertes Screening/Diagnostik,
4. (ursächliche und symptomspezifische) Behandlung aller Formen von Delirien und
5. Überleitung, Verlaufsbeobachtung und Beendigung, insbesondere der pharmakologischen Behandlung.

### Risiko- und modifizierbare Faktoren eines Delirs einbeziehen

Delirien sind in der älteren Bevölkerung mit Prävalenzen bis zu 70 % eine der Hauptursachen für vermeidbare Erkrankungen bei älteren Krankenhauspatienten [25]. Die Häufigkeit von Delirien in der Krankenhausbehandlung variiert ebenso stark (< 20 und > 70 %; [26]).

Vier der wichtigsten nicht veränderbaren Faktoren sind höheres Alter, kognitive Beeinträchtigung, Schwere der zugrunde liegenden medizinischen Erkrankung und bereits bestehende psychische Störungen. Zu den zahlreichen potenziell veränderbaren Risikofaktoren, die durch frühzeitiges Eingreifen und/oder Prävention beeinflussbar sein können, gehören: die Einnahme verschiedener pharmakologischer Wirkstoffe (insbesondere GABAerge und Opioidwirkstoffe sowie Medikamente mit anticholinerger Wirkung), verlängerte und/oder ununterbrochene Sedierung, Immobilität, Substanzintoxikation und -entzug, die Anwendung physischer Zwangsmaßnahmen, Wasser- und Elektrolytstörungen, Ernährung bzw. Nährstoffmängel, Stoffwechselstörungen und Endokrinopathien (vor allem Mangel oder Überschuss an Kortisol, Stress), schlechte Sauerstoffversorgung (z. B. Hypoperfusion, Hypoxämie, Anämie), fehlende (Nacht‑)Ruhe (Licht, Monitorgeräusche und Untersuchungen nachts) oder gestörter Schlaf-Wach-Rhythmus sowie Schmerzen.

### Diagnostische Herausforderung

Trotz der Häufigkeit bleiben fast paradoxerweise delirante Zustände oft unerkannt (Angaben bis über 50 %). Angesichts des fluktuierenden Verlaufs mit v. a. Tag/Nacht-Schwankungen ist es erforderlich, dass eine umfassende konsiliarische psychiatrische Beurteilung alle verfügbaren klinischen Informationen berücksichtigt, gerade auch der Befragung von Familienmitgliedern und Pflegekräften sowie einer Überprüfung der Krankenakte auf Verhaltensweisen, die über 24 h, auch vor der klinischen Untersuchung, aufgetreten sind.

Es gibt zahlreiche validierte Messinstrumente [10, 27], welche settingspezifisch (Checklisten und klinische Tests, gerade auch für Pflegeteams) angewandt werden können. Deren Einsatz sollte strukturiert erfolgen, z. B. postoperativ oder in ITS-Behandlung regelmäßig pro Schicht oder im geriatrischen Setting sowie in der Rettungsstelle. Der Stellenwert erweiterter Diagnostik (z. B. Labor und Bildgebung) liegt vor allem in der Ursachensuche und der erweiterten oder Verlaufsdiagnostik sowie in der differenzialdiagnostischen Abgrenzung anderer neurologischer Erkrankungen (z. B. nichtkonvulsiver Status).

### Nichtpharmakologische Behandlungsmaßnahmen

Propagiert werden einfache Maßnahmen, die vom Krankenhauspersonal angewendet werden können. Dazu zählen in erster Linie eine Unterstützung der (Neu‑)Orientierung, angemessene kognitive Stimulation tagsüber, die Umsetzung eines (nichtpharmakologischen) Schlafhygieneprotokolls zur Normalisierung des Schlaf-Wach-Rhythmus, frühe Mobilisierung nach einer Operation oder Extubation [9].

Außerdem sind dies die rechtzeitige Entfernung von Kathetern und Fixierungen, frühzeitige Korrektur sensorischer Defizite (z. B. Brillen und Hörgeräte) und von Dehydration und Elektrolytstörungen und auch Lichttherapie.

Bemerkenswert ist, dass schon die Durchführung der Interventionen durch nichtprofessionelle Familienmitglieder zu einer signifikanten Reduktion von Delirien führte. Interessanterweise können bereits einfache Intervention (z. B. tägliche Neuorientierung, ergänzt durch Umwelt-, akustische und visuelle Stimulation) das Auftreten von Delirien signifikant senken [28]. Programme durch Laienhelfende erscheinen in spezifischen Abteilungen vor diesem Hintergrund durchaus sinnvoll (z. B. Delirlotsen in der Ersten Hilfe, [29]). Richtlinien für Interventionen und Übersichtsempfehlungen basieren mittlerweile maßgeblich auf den nonpharmakologischen Faktoren [10].

### Pharmakologisch Behandlung

Daten, welche auch prospektiv und in Teilen randomisiert und doppelblind zur Vorbeugung oder Behandlung von Delirien erhoben wurden, deuten darauf hin, dass pharmakologische Strategien (z. B. Haloperidol, Antipsychotika der 2. Generation, Gabapentin, Melatonin, eine Einzeldosis Ketamin während der Narkoseeinleitung und eine auf Dexmedetomidin basierende Sedierung) bei der Vorbeugung von Delirien erfolgreicher waren als bei seiner Behandlung [30, 31]. Die Effekte der unterschiedlichen Substanzklassen sollten deshalb differenziert symptombezogen und mit den jeweiligen unerwünschten Wirkungen abgewogen werden.

Gerade im geriatrischen Bereich ist hier besondere Vorsicht, ob der möglichen unerwünschten Wirkungen geboten und das Absetzen nach Beendigung der Behandlung explizit zu erwähnen [32]. Ein Check der Interaktionen und Empfehlungen der Reduktion von Polypharmazie können hier auch erfolgen. Es kann sich auch lohnen, mit einzelnen unterschiedlichen Fachabteilung, gerade wenn eine gewisse Spezialisierung im Setting und in der behandelten Klientel vorliegt, Behandlungsregime gemeinsam zu formulieren.

In der Psychopharmakotherapie von Delirien ist zu beachten, dass die Evidenz und Leitlinienempfehlung schwach oder nicht vorhanden ist [4]. Gerade bei M. Parkinson, demenziellen Erkrankungen und im Senium ist bei Antipsychotika in der Anwendung Vorsicht geboten. Acetylcholinesterasehemmer verbleiben ohne klare Evidenz nicht empfohlen zur Delirprävention bei außerdem eingeschränkter Verträglichkeit.

#### Infobox Information zum Thema Delir

- European Delirium Association: https://www.europeandeliriumassociation.org
- Nature Disease Reviews Primers Delirium: https://www.nature.com/articles/s41572-020-00223-4
- AWMF(Arbeitsgemeinschaft der Wissenschaftlichen Medizinischen Fachgesellschaften)-S3-Leitlinie Analgesie, Sedierung und Delirmanagement in der Intensivmedizin: https://register.awmf.org/de/leitlinien/detail/001-012Leitlinie Anästhesie
- AWMF-S1-Leitlinie Delir und Verwirrtheitszustände inklusive Alkoholentzugsdelir: https://register.awmf.org/de/leitlinien/detail/030-006
- Aktualisierte ESAIC(European Society of Anaesthesiology and Intensive Care)-Leitlinie POST-OP Delir: https://www.springermedizin.de
- Empfehlungen zum Delirmanagement aus der Nationalen Demenzstrategie: https://www.dgppn.de

## Fazit für die Praxis

- Delirien führen häufig zu dringenden CL(psychiatrische Konsil- und Liaisondienst)-Anfragen, folgerichtiger ist ein abteilungsspezifisches Delirmanagement.
- Gerade auch geriatrische Risikofaktoren sollten systematisch erfasst und kontrolliert werden (Polypharmazie, Interaktionen, Anticholinergika, Sedativa).
- Im CL kann die strukturierte Detektion, Ursachensuche und spezifische Behandlung und deren Überleitung unterstützt werden.
- Nichtpharmakologische Maßnahmen sollten mit den Teams besprochen werden.
- Pharmakotherapie sollte zurückhaltend abgewogen und differenziert symptombezogen empfohlen werden (Beendigung nicht vergessen!).
- Die Kommunikation im multiprofessionellen Team und mit den Betroffenen und Angehörigen sollte gezielt (wertfrei, empathisch zugewandt) unterstützt werden.
- Interdisziplinäre In-house-Weiterbildung kann durch den CL unterstützt werden.
- Es existieren aktuelle und differenzierte Leitlinien und fachliche Empfehlungen.
